# Impact of intradialytic fiber clotting on dialyzer extraction and solute removal: a randomized cross-over study

**DOI:** 10.1038/s41598-022-09696-7

**Published:** 2022-04-05

**Authors:** Floris Vanommeslaeghe, Iván Josipovic, Matthieu Boone, Wim Van Biesen, Sunny Eloot

**Affiliations:** 1 Nephrology Department, Ghent University Hospital, Corneel Heymanslaan 10, 9000 Gent, Belgium; 2 Centre for X-Ray Tomography, Physics and Astronomy, Ghent University, Ghent, Belgium

**Keywords:** Nephrology, Renal replacement therapy

## Abstract

Previous studies revealed the importance of biocompatibility, anticoagulation strategy, and dialysis mode and duration on fiber blocking at the end of a hemodialysis session. The present study was set up in ten hemodialysis patients to relate fiber patency to dialyzer extraction and removal of small and middle molecules. With only 1/4th of the regular anticoagulation dose, and using a Solacea 19H and FX800 CorDiax dialyzer, fiber patency was quantified using 3D micro-CT scanning for different dialysis durations (i.e. 60, 120 and 240 min). While Solacea showed enhanced fiber patency in all test sessions, fiber blocking in the FX800 CorDiax did not follow a linear process during dialysis, but was rather accelerated near the end of dialysis. Dialyzer extraction ratios were correlated with the percentages of open fibers. While the fiber blocking process affected extraction ratios (i.e. for phosphorus and myoglobin in the FX800 CorDiax), it had only minor impact on the removal of toxins up to at least 12 kDa.

## Introduction

During hemodialysis, the use of anticoagulation is standard of care to prevent clotting of the extracorporeal circuit^1^. Anticoagulation needs to be well-balanced to avoid an increased risk for bleeding complications on the one hand, and clotting of the extracorporeal circuit resulting in blood loss for the patient on the other hand.

Previous research of our group using the gold standard of fiber quantification by micro-CT indicated that a substantial number of fibers can become blocked before this is reflected in a change of routinely observed dialysis parameters or in termination of the dialysis session^2^. We also demonstrated that type of dialyzer (more or less biocompatible), dose and type of anticoagulation strategy, and duration of the dialysis session, next to patient factors such as inflammation, impact substantially on the number of patent fibers at the end of the dialysis session^3–8^.

This study aims to answer two major remaining questions: the kinetics of fiber blocking during the dialysis session, and the degree to which unnoticed fiber blocking impacts overall dialyzer performance in terms of extraction ratio and total solute removal.

Dialyzer extraction ratios for the middle molecules myoglobin and lambda and kappa free light chains, as measured at 1 h after dialysis start, were not found associated with dialyzer fiber patency post dialysis^3^. This absence of association suggests that fiber blocking is a more delayed phenomenon that occurs only later in the dialysis session. If this would be confirmed, the impact on total solute removal would be limited, as most of the capacity for solute removal of the membrane would still be available during most of the dialysis session.

To investigate the potential impact of fiber blocking on solute clearance in a more robust and representative manner, extraction ratios should be measured just before the dialyzer is disconnected from dialysis and further prepared for micro-CT scanning. Applying such a strategy after different durations of dialysis time would not only allow to evaluate the kinetics of percentage fiber blocking over the dialysis session, but also to associate this with the impact on solute clearance and removal.

## Results

Relevant demographic and clinical data of the patient population at baseline (age 57.0 ± 10.2; all male) are summarized in Table 1. There were no patient dropouts during the experimental period, scheduled dialysis duration and flow settings were maintained according to the protocol in all the test sessions, and no adverse events were recorded.

**Table 1:** Demographic and clinical data of the patient population at baseline.

Gender (M/F)	10 M
Age (years)	57.0 ± 10.2
Dry body weight (kg)	74.7 [69.7;89.1]
Dialysis vintage (months)	23.1 [12.5;40.9]
Renal disease	Autosomal dominant polycystic kidney disease (*n* = 3); IgA nephropathy (*n* = 2); diabetic nephropathy (*n* = 1); reflux nephropathy (*n* = 1); focal segmental glomerulosclerosis (*n* = 1); membranous glomerulonephritis (*n* = 1); Alport (*n* = 1)
Regular anticoagulation dose	Enoxaparin 40 mg (*n* = 5); Tinzaparin 3500 (*n* = 4); Enoxaparin 60 mg (*n* = 1)
Platelet inhibitors	Acetylsalicylic acid 80 mg (*n* = 7)
Hb (g/dL)	12.0 ± 0.7
Platelets count (10^3^/µL)	224 ± 56
aPTT (s)	34.6 ± 2.6
INR (-)	1.0 ± 0.1
CRP (mg/L)	5.2 [2.3;7.0]

Hb: hemoglobin; aPTT: activated partial thromboplastin time; INR: international normalized ratio; CRP: C-reactive protein.

**Figure 1:**
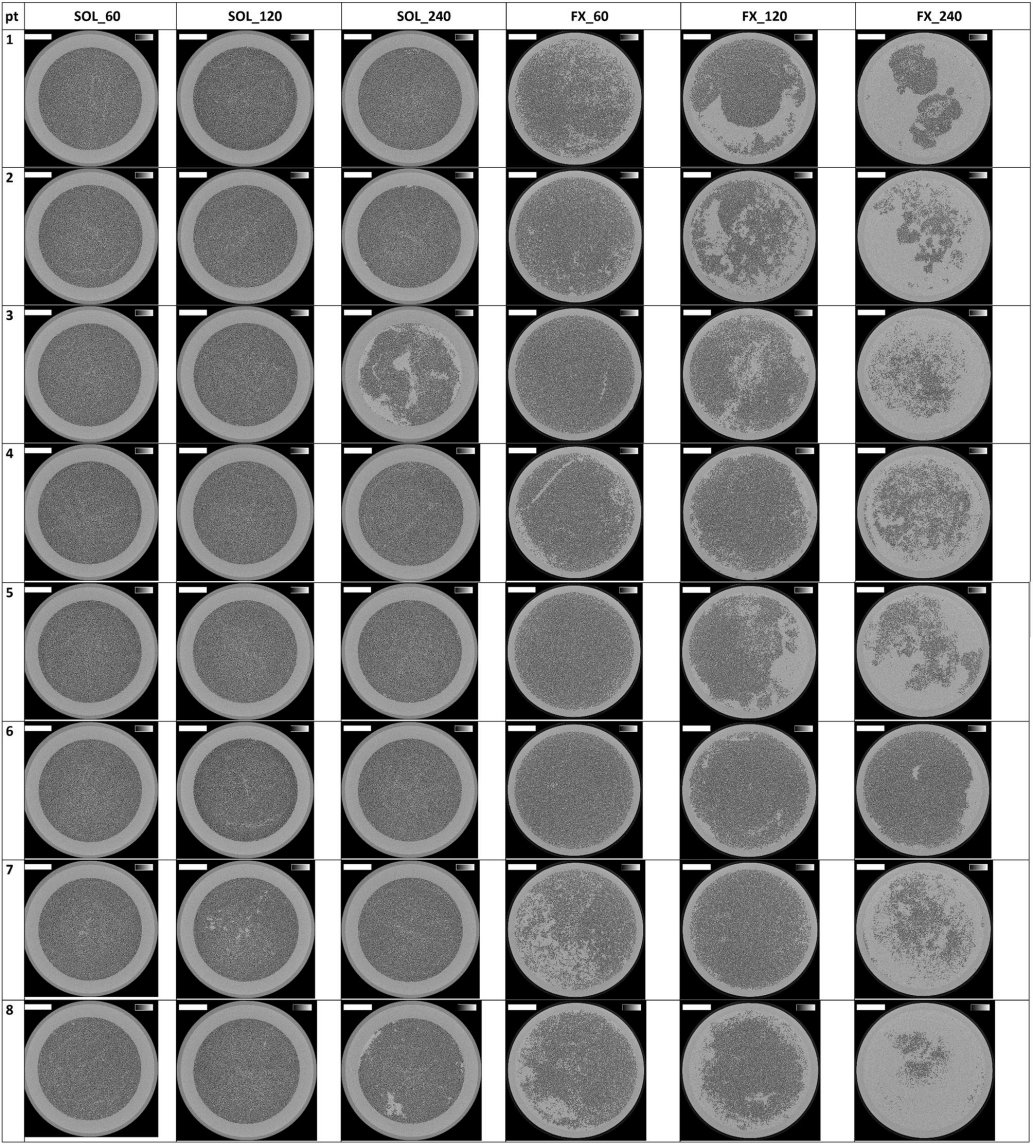

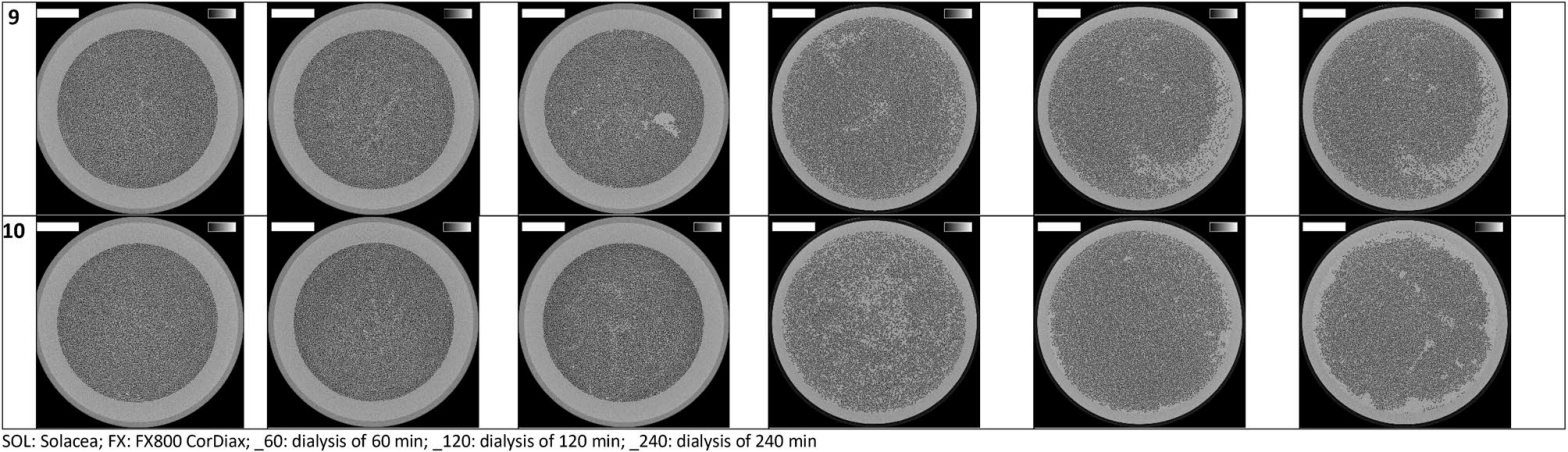
Cross-sections halfway the outlet potting in 10 patients, 2 dialyzers and for each 3 dialysis durations. The greyscale range is from 0 to 0.5 cm^-1^ and the scale bar denotes 10 mm.

Cross-sections halfway the dialyzer outlet potting for all the six experimental dialysis sessions of each patient are presented in Fig. 1. The lumens of open fibers are visualized as black dots. The number of open fibers in the three non-used Solacea samples was 12,095 ± 1 and in the non-used FX800 CorDiax dialyzers 13,047 ± 1. In Table 2 this number is compared for both types of dialyzers in the six test sessions, using three different thresholds to define the surface area of an open fiber: i.e. 50, 70 and 90% of the cross-section of a non-used fiber.

**Table 2:** Percentage of open fibers in the six test sessions (*n* = 10) for the thresholds of 50, 70 and 90% open fiber area.

Open fiber area (%)	SOL_60	SOL_120	SOL_240	FX_60	FX_120	FX_240	Friedman *P*-value
50	100 [100;100]	100 [100;100]	99 [98;100]	90 [81;98]^α β^	84 [69;92]^α β^	32 [27;43]^α β γ^	< 0.001
70	100 [99;100]	100 [99;100]	99 [97;99]	90 [81;98]^α β^	83 [68;92]^α β^	31 [26;41]^α β γ^	< 0.001
90	74 [70;79]	74 [67;88]	64 [59;69]	63 [56;65]	52 [38;59]^α β^	14 [9;17]^α β γ^	< 0.001

median [25pct;75pct]; SOL: Solacea; FX: FX800 CorDiax; _60: dialysis of 60 min; _120: dialysis of 120 min; _240: dialysis of 240 min.

^α^*P* < 0.05 versus SOL_60; ^β^P < 0.05 versus SOL_120; ^γ^P < 0.05 versus SOL_240.

**Figure 2:**
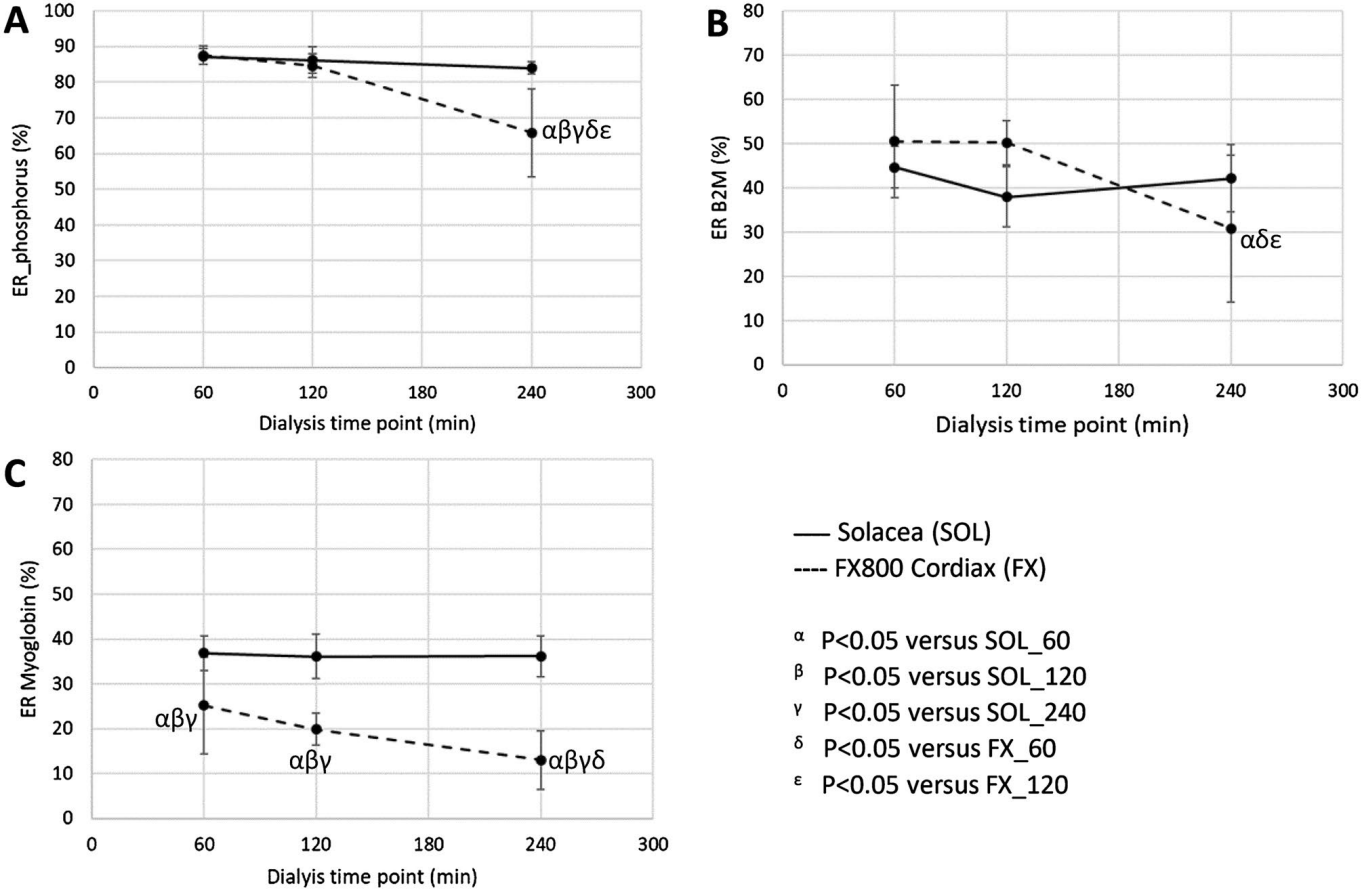
Extraction ratio (ER) of phosphorus (panel **A**), beta-2-microglobulin (panel **B**—B2M) and myoglobin (panel **C**) as measured in 10 patients at the end of a dialysis session with different durations (i.e. 60, 120 and 240 min) and with the Solacea (full line) and the FX800 CorDiax dialyzer (dashed line) (*n* = 10).

Extraction ratios (ERs) in the Solacea and FX800 CorDiax for phosphorus, β2M, and myoglobin are presented in Table 3 and Fig. 2 for the different dialysis durations. End dialysis ERs for the investigated solutes are equal for the three different dialysis durations in the Solacea dialyzer, while in the FX800 CorDiax they were substantially lower at 240 min versus 60 min dialysis. Furthermore, phosphorus and myoglobin ERs at 240 min dialysis in the FX800 CorDiax were also lower than the ERs in the Solacea at any measured time point.

**Table 3:** Extraction ratios for phosphorus, β_2_M and Myoglobin in the six dialysis test sessions (*n* = 10).

ER (%)	SOL_60	SOL_120	SOL_240	FX_60	FX_120	FX_240	Anova *P*
Phosphorus	87 ± 2	86 ± 4	84 ± 2	88 ± 3	85 ± 3	66 ± 12^αβγδε^	< 0.001
β_2_M	45 ± 5	38 ± 7	42 ± 8	51 ± 13	50 ± 5	30 ± 17^αδε^	< 0.001
Myoglobin	37 ± 4	36 ± 5	36 ± 5	25 ± 11^αβγ^	20 ± 4^αβγ^	13 ± 7^αβγδ^	< 0.001

β_2_M: beta-2-microglobulin; SOL: Solacea; FX: FX800 CorDiax; _60: dialysis of 60 min; _120: dialysis of 120 min; _240: dialysis of 240 min.

^α^*P* < 0.05 versus SOL_60; ^β^P < 0.05 versus SOL_120; ^γ^P < 0.05 versus SOL_240; ^δ^P < 0.05 versus FX_60; ^ε^P < 0.05 versus FX_120.

**Figure 3:**
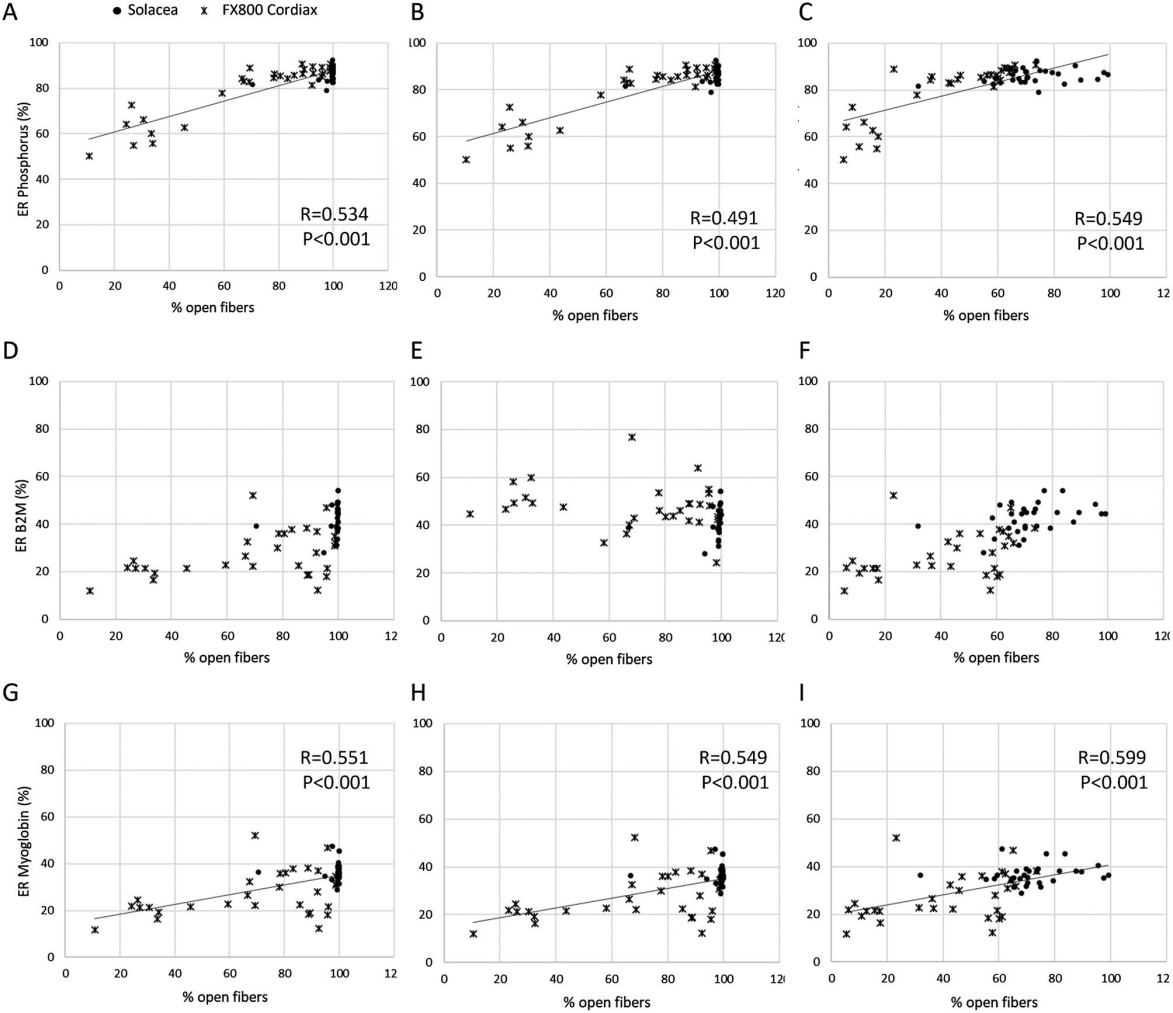
Relations between ER and % open fibers at the end of dialysis with the Solacea (dots) and the FX800 CorDiax (crosses) for phosphorus (panels **A**–**C**), B2M (panels **D**–**F**), and Myoglobin (panels **G**–**I**) for different % of open fiber area: 50% (panels **A**, **D**, **G**), 70% (Panels **B**, **E**, **H**), and 90% (panels **C**, **F**, **I**).

The relations between the ER as measured at the end of the dialysis session and the visualized percentage of open fibers are shown in Fig. 3 for the different percentages of open fiber area (50, 70 and 90%). Due to the enhanced fiber patency observed in the Solacea dialyzer, there is a lack of variation in % open fibers and, as a consequence, no correlation can be seen for Solacea alone. Combining the results of all test sessions with Solacea and FX800, correlations with % open fibers were found for phosphorus and myoglobin.

**Figure 4:**
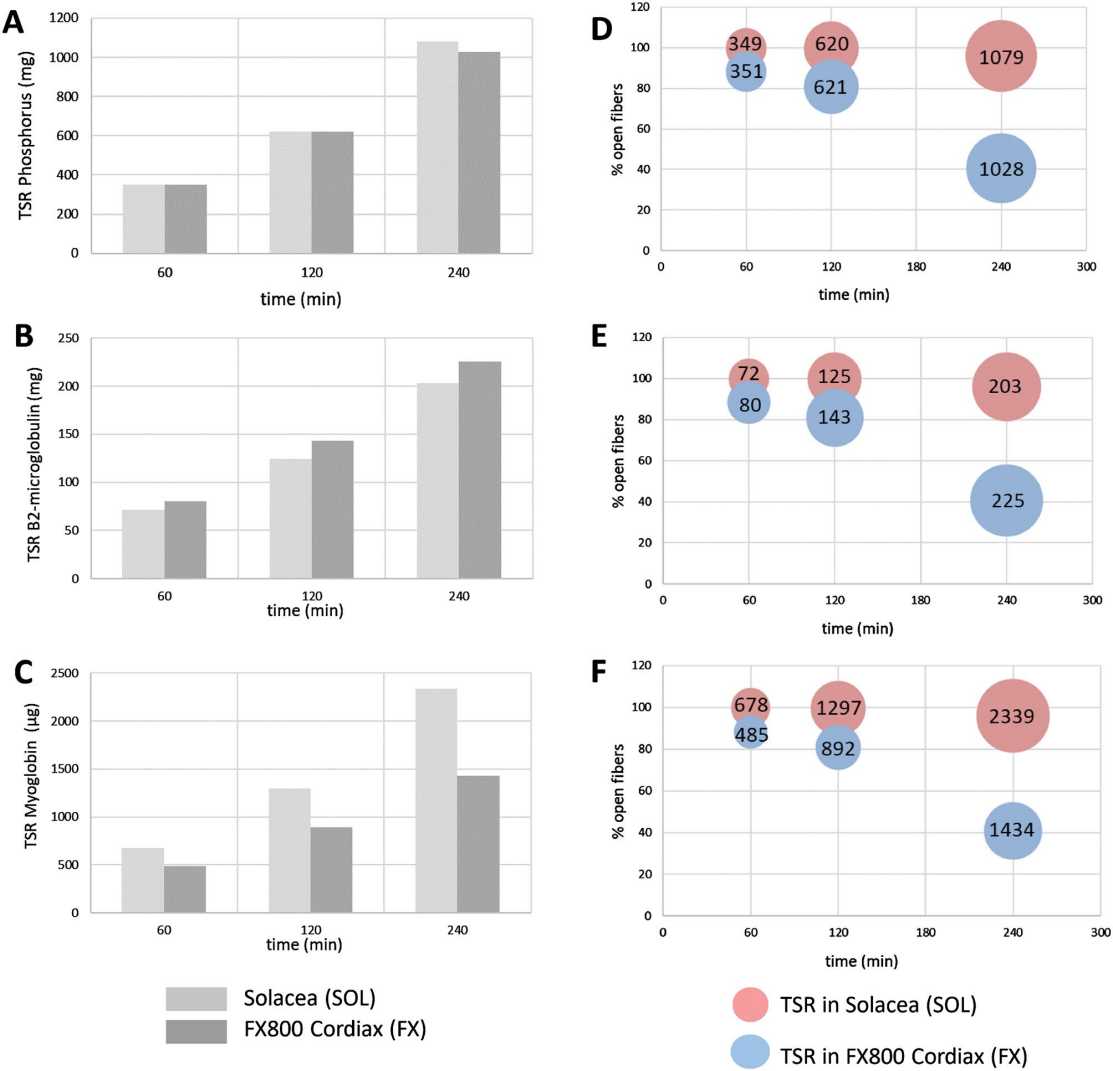
Total Solute Removal as theoretically calculated for a virtual patient, cumulated during dialysis (panels **A**–**C**), and as an extra function of the percentage open fibers (panels **D**–**F**) for phosphorus (panel **A** + **D**—mg), β2-microglobulin (panel **B** + **E—**mg), and Myoglobin (panel **C** + **F—**µg).

The theoretically calculated cumulative Total Solute Removals (TSR) during the course of a dialysis session of 240 min, and this for the three solutes and two dialyzers under study, are shown in Fig. 4 (panels A–C) and as an extra function of the percentage open fibers in Fig. 4 (panels D–F).

## Discussion

To our knowledge, this is the first study to directly link fiber blocking with dialyzer performance in terms of extraction and total solute removal. This cross-over study investigated fiber blocking and dialyzer extraction ratios at different dialysis time points in the Solacea and FX800 CorDiax dialyzers, during post-dilution hemodiafiltration with reduced anticoagulation dose. It was demonstrated, based on data of three time points, that kinetics of fiber blocking during dialysis is not a linear process, but is accelerated during the second half of dialysis. Furthermore, dialyzer ERs for phosphorus and myoglobin were correlated with the percentages of open fibers, and were consequently lower with the FX800 CorDiax versus Solacea. Last, the large increase in fiber blocking in the FX800 CorDiax during the last part of the dialysis sessions had only minor influence on total solute removal of phosphorus and the middle molecule β_2_M, while myoglobin solute removal was hampered.

Fiber patency in the Solacea dialyzer remained optimal until the end of the 240 min dialysis session for 50% and even for 70% open fiber area, while, mainly due to deposition onto the membrane, fiber patency was 74% (at 60 min) down to 64% (at 240 min) when only counting the 90% open fibers. Apart from this thin layer, percentage open fibers in the FX800 CorDiax was already lower after a 60 min dialysis, and was, for 70% open fiber area, further decreased by 66% (interval 60–240 min) and 63% (interval 120–240 min). These data confirm earlier work, in which minimal activation of the coagulation cascade was observed in the Solacea dialyzers, even at very low levels of anticoagulation^3,5,9^.

We also observed that fiber blocking is not a smooth process with linear kinetics over time, but rather follows an exponential pattern, accelerating progressively in the later stages of the dialysis session. Our current study setup unfortunately does not allow a minute-by-minute analysis of the coagulation process, as the gold standard micro-CT analysis requests that the dialysis session should be stopped. Nevertheless, our data do support a rather limited activation of coagulation in the first 120 min of the dialysis session, despite very low application of anticoagulant, and a more accelerated activation thereafter based on measurements at 210 and 240 min dialysis. Furthermore, in the Solacea dialyzers, even after 240 min, fiber blocking was nearly completely absent even when the cut-off is set at a minimal decrease of fiber diameter. This suggests, or is at least compatible with the hypothesis that coagulation is an on/off phenomenon and that once the cascade is activated, there is a rather rapid evolution to complete fiber blocking. However, to our knowledge, accurate tools to quantify fiber blocking online are currently not available to confirm or contradict this hypothesis.

Although a correlation was found between extraction ratio and percentage open fibers, substantial decrease in fiber patency did not result in a proportional decrease in solute extraction. Furthermore, whereas extraction rate decreased by 25% for the small solute phosphorus, and even by 41 and 48% for the middle molecules β_2_M and myoglobin, mathematical analysis showed relatively modest decreases in total solute removal rates of only 6, 7 and 9% for phosphorus, β_2_M and myoglobin, respectively, during the dialysis period 120–240 min. This can be explained by the fact that the instant absolute solute removal is always a proportion of the solute concentration at the dialyzer inlet, and is thus not only determined by the extraction ratio of the dialyzer. Since inlet concentrations are declining during dialysis according to single or multicompartmental solute kinetics in the patient’s body, the absolute solute removal will also decline over time. Because fiber blocking was found to be non-linear and most accelerated during the second part of dialysis, the impact of fiber blocking near the dialysis end has a rather limited impact on overall solute removal. This effect is the smallest for those solutes with the highest reduction ratio during dialysis (i.e. with the largest decline from pre to post dialysis concentration), e.g. small water soluble solutes like urea and creatinine^10^. As a result, evaluation of dialyzer coagulation based on urea removal is not representative for fiber patency. The fact that urea clearance might not be the ideal parameter to evaluate dialyzer clotting, is endorsed by previous K∙t/V_urea_ based studies, which did not find significances among different dialysis strategies, while visual inspection and premature termination of the session did^11,12^.

Phosphorus is known to have complex kinetics in the patient, characterized by mobilization of solute transport from the deeper tissues towards the blood, once plasma levels are decreased^13,14^. This results in rather constant or even increased dialyzer inlet concentrations during the second half of dialysis, giving the potential of high solute removal in case extraction would have remained constant. Theoretical calculations accordingly reveal that the hampered extraction in the FX800 CorDiax contributed to a loss in total solute removal of only 6.6%. For the more patent Solacea dialyzer, this loss in total solute removal is even more negligible, and limited to only 0.9%.

Middle molecules like β_2_M are known to have retarded transport within the patient, a property that plays a more prominent role in solute removal than dialyzer extraction ratio does^15–17^. This implies that for solutes behaving like β_2_M, absolute solute removal is less sensitive to ad hoc changes in extraction. Accordingly, we can explain why the loss in solute removal remained limited to a theoretically calculated 5.6% (Solacea) and 7.6% (FX800 CorDiax), despite observed large differences in extraction ratio at the end of the dialysis session between the two filters. A further reason why both dialyzers perform in the same range is the cross-over in β_2_M extraction (Fig. 2B). The somewhat larger sieving coefficient for β_2_M in the FX800 CorDiax (0.85 versus 0.8 in the Solacea) explains the higher extraction during the first half of dialysis, while the higher degree in fiber blocking during the second half decreases the dialyzer extraction.

For the middle molecule myoglobin, calculations suggest a 16.5% loss in total solute removal in the FX800 CorDiax due to the decreasing ER during dialysis. The overall lower myoglobin removal as compared to removal in Solacea was determined by the lower sieving coefficient (0.5 in FX800 CorDiax versus 0.8 in Solacea) from the start of dialysis on, and the lower extraction ratio towards the end of dialysis due to fiber blocking. The patent fibers in the Solacea make this dialyzer superior for the removal of large middle molecules like myoglobin, with a calculated loss in TSR of only 0.9%.

Our study reveals that in routine clinical practice, many dialysis patients might still receive supra-therapeutic doses of anticoagulation for their dialysis session. Indeed, despite giving only a quarter of the regular anticoagulation dose, all sessions could be completed successfully. As bleeding complications are still prevalent in chronic dialysis patients, there is room for further optimization of the anticoagulation regime. However, such an optimization is hampered by the lack of an adequate tool to quantify coagulation online during a dialysis session. Complete blocking of the circuitry as a sign of insufficient anticoagulation is a clear and intrusive event, often leading to a permanent increase in dose of anticoagulation. However, over-anticoagulation is less easily detected and therefore, most likely, dose reductions in case of overdosed anticoagulation occur less frequently.

In order to evaluate fiber blocking kinetics, we had to perform separate dialysis sessions of different duration rather than measuring at different time points in a single session, which could be considered a limitation of the study. However, this is the only way to go when using the very sensitive micro-CT scanning technique, the gold standard to quantify dialyzer fiber blocking^2,18^. In addition, patients were their own controls, and to have the best and similar cross-over starting conditions, group randomization was performed per dialyzer.

As a strength of our study, we were using the same type of dialysis strategy and the same amount of anticoagulant administered always in an identical way (i.e. single bolus at the dialysis start) for all dialysis sessions. This allowed us to make a direct comparison between the dialyzers and between the sessions of different duration.

In conclusion, this study confirmed that the Solacea dialyzer is more resistant to activation of coagulation. Fiber blocking was found to be a non-linear process which is accelerated during the second part of dialysis based on measurements at three time points. While fiber blocking seemed to have only a minor impact on the removal of toxins up to at least 12 kDa, the removal of larger toxins like myoglobin was found hampered by fiber blocking.

## Patients and methods

### Patients

This single center cross-over study (May–June 2021) included ten stable chronic hemodialysis (HD) patients who had stable dialysis sessions during the last 4 weeks, and were not known to have a coagulation disorder, active inflammation or malignancy. Double-needle vascular access was achieved through a native arterio-venous fistula.

Power analysis was based on the relative number of patent fibers in a previously performed cross-over study in 10 patients. Power was at least 98% (α = 0.05) using two different types of dialyzers^3^.

The protocol adhered to the Declaration of Helsinki, was approved by the institutional research committee (Ethical Committee—Ghent University Hospital, BC 08511—B6702020000758—11/2020), and was registered in www.clinicaltrials.gov (NCT04746391—09/02/2021—‘Impact of Clotting on Dialyzer Efficiency’). Written informed consent was obtained from all included patients.

### Dialysis and anticoagulation

In the study protocol, each patient was dialyzed with six different regimens at midweek, with three different dialysis durations, i.e. 60, 120, and 240 min, and two different types of dialyzers, i.e. the ATA Solacea 19H dialyzer (Nipro, Osaka, Japan) and the FX800 CorDiax (Fresenius Medical Care, Bad Homburg, Germany).

To amplify the coagulation process, patients received only a quarter of their regular dose Low-Molecular-Weight Heparin at the dialysis start (i.e. enoxaparin Clexane 40 mg, Sanofi, Belgium).

All test sessions were performed on a 5008 dialysis machine (Fresenius Medical Care, Bad Homburg, Germany) in post-dilution hemodiafiltration with blood flow 300 mL/min, dialysate flow 500 mL/min, and substitution at 25% of blood flow (i.e. 75 mL/min). Ultrafiltration rates were set according to the patient’s interdialytic weight gain and clinical status.

Group randomization (https://www.randomizer.org/) was performed (by the study coordinator) per dialyzer for the three dialysis durations 60, 120, and 240 min (Table 4). Each experimental session was preceded with two wash-in sessions using the same type of dialyzer as in the experimental dialysis at midweek, but always with full regular anticoagulation dose. Each patient served as his/her own control.

**Table 4:** Test protocol.

1/4 anticoagulation (*n* = 10)					
6 test sessions					
Solacea 19H			FX800CorDiax		
Group randomization			Group randomization		
Post-HDF	Post-HDF	Post-HDF	Post-HDF	Post-HDF	Post-HDF
60 min	120 min	240 min	60 min	120 min	240 min
SOL_60	SOL_120	SOL_240	FX_60	FX_120	FX_240

Post-HDF: post dilution hemodiafiltration.

Apart from the study protocol, following the study sessions of only 60 and 120 min, patients were dialyzed with a new dialysis circuit for another 180 and 120 min, respectively, to ensure adequate dialysis.

### Blood sampling, laboratory and calculations

At the end of each of the experimental session, blood was sampled from the dialyzer inlet and outlet blood lines. Blood samples were immediately centrifuged and serum was stored at −80 °C until batch analysis. Concentrations of the small water-soluble solute phosphorus (molecular weight 31 Da) were determined by routine analysis, while those of the middle molecules beta-2-microglobulin (β2M—11 kDa) and myoglobin (17 kDa) were, respectively, determined by ELISA and nephelometry. Colorimetric bromocresol green assay was used to determine Albumin (Alb) concentrations. All analyses were performed in the Routine Laboratory of the Ghent University Hospital, apart from β2M for which analyses were done in the Laboratory of the Nephrology Department (Ghent University Hospital).

Dialyzer Extraction Ratios (ER—%) were calculated from the inlet and outlet serum concentrations (C_inlet_ and C_outlet_), and corrected for hemoconcentration (based on Alb levels) for the middle molecules:

1 $$ {\text{ER}}(\% ) = \frac{{{C_{{\text{inlet}}}} - {C_{{\text{outlet}}}} \cdot \left( {{\raise0.7ex\hbox{${Al{b_{{\text{inlet}}}}}$} \!\mathord{\left/ {\vphantom {{Al{b_{{\text{inlet}}}}} {Al{b_{{\text{outlet}}}}}}}\right.\kern-\nulldelimiterspace}\!\lower0.7ex\hbox{${Al{b_{{\text{outlet}}}}}$}}} \right)}}{{{C_{{\text{inlet}}}}}} \cdot 100$$

Total Solute Removal (TSR) was calculated as the area under the curve of the calculated concentration in spent dialysate. To compare mass removal among different dialysis sessions with different dialyzers and different start concentrations, a mean inlet serum concentration was used per time point (60, 120, and 240 min) and start concentrations (0 min) were derived from extrapolation of the slope of the concentration decrease between time point 60 and 120 min. From those theoretical inlet serum concentrations, outlet serum concentrations (C_outlet_) were derived based on the Extraction Ratio (Eq. 1) at the different time points (60, 120, and 240 min), and dialysate outlet concentrations (C_dialysate_) were derived from the mass balance in the dialyzer as a function of the inlet concentration (C_inlet_), plasma flow (Q_plasma_) as calculated accounting for the hematocrit, dialysate flow (Q_dialysate_), and ultrafiltration rate (Q_UF_):

2 $$ {C_{{\text{inlet}}}} \cdot {Q_{{\text{plasma}}}} = {C_{{\text{outlet}}}} \cdot \left( {{Q_{{\text{plasma}}}} - {Q_{{\text{UF}}}}} \right) + {C_{{\text{dialysate}}}} \cdot \left( {{Q_{{\text{dialysate}}}} + {Q_{{\text{UF}}}}} \right)$$

3 $$ {C_{{\text{dialysate}}}} = \frac{{{C_{{\text{inlet}}}} \cdot {Q_{{\text{plasma}}}}\; - \;{C_{{\text{outlet}}}} \cdot \left( {{Q_{{\text{plasma}}}}\; - \;{Q_{{\text{UF}}}}} \right)}}{{{Q_{{\text{dialysate}}}} + \;{Q_{{\text{UF}}}}}}$$

### Micro-CT scanning and coagulation quantification

At the end of the study session, a standard rinsing procedure of the hemodialyzer was performed with exact 300 mL rinsing solution. Next, the hemodialyzer was dried for 12 h applying continuous mild positive pressure ventilation, simultaneously in blood and dialysate compartment. Dialyzer fiber blocking was visualized in the dialyzer outlet potting using a reference micro-CT scanning technique^2,18^.

Octopus Reconstruction software package as licensed by XRE, a Ghent University spin-off company, is used to reconstruct the raw projection data into 2D visualizations. Non-blocked fibers were counted in the central cross-section of the dialyzer outlet potting, using an open-source platform for biological-image analysis (ImageJ 1.51 H, NIH, Bethesda, USA). Three different thresholds were used to define the surface area of an open fiber: i.e. 50, 70, and 90% of the cross-section of a non-used fiber. Comparing the number of non-blocked fibers in the tested dialyzer with the total number of fibers as measured in three non-used dialyzer samples, provides an objective estimate of the percentage of fiber blocking.

### Statistical analysis

Statistical analyses were performed using SPSS (version 26, SPSS Inc, Chicago, USA). Continuous variables were summarized as mean ± SD or median [25th percentile; 75th pct]. To compare different related variables, ANOVA tests were performed with Bonferroni post hoc test (normal distributions) or Friedman tests with Wilcoxon post hoc test (no normal distributions). To relate different parameters, Spearman correlations were performed.

## Data availability

All data analyzed during this study are included in this published article.
